# Cellular Therapy in NSCLC: Between Myth and Reality

**DOI:** 10.1007/s11912-023-01443-z

**Published:** 2023-08-30

**Authors:** Martina Imbimbo, Laureline Wetterwald, Alex Friedlaender, Kaushal Parikh, Alfredo Addeo

**Affiliations:** 1 Oncology Department, Centre Hospitalier Universitaire Vaudois (CHUV), Rue du Bugnon 46, Lausanne University Hospital, Lausanne, Switzerland; 2 Oncology Department, University Hospital Geneva (HUG), 1205 Geneva, Switzerland; 3 Oncology Department, Clinique Générale Beaulieu, 1206 Geneva, Switzerland; 4 Division of Medical Oncology, Mayo Clinic, Rochester, MN USA

**Keywords:** Adoptive cell therapies, NSCLC, Engineered adoptive cell therapies, TCR, CAR-T, Tumor-infiltrating lymphocytes (TILs)

## Abstract

**Purpose of Review:**

In this paper, we review the current state and modalities of adoptive cell therapies (ACT) in non-small cell lung carcinoma (NSCLC). We also discuss the challenges hampering the use of ACT and the approaches to overcome these barriers.

**Recent Findings:**

Several trials are ongoing investigating the three main modalities of T cell-based ACT: tumor-infiltrating lymphocytes (TILs), genetically engineered T-cell receptors (TCRs), and chimeric antigen receptor (CAR) T cells. The latter, in particular, has revolutionized the treatment of hematologic malignancies. However, the efficacy against solid tumor is still sparse. Major limitations include the following: severe toxicities, restricted infiltration and activation within the tumors, antigen escape and heterogeneity, and manufacturing issues.

**Summary:**

ACT is a promising tool to improve the outcome of metastatic NSCLC, but significant translational and clinical research is needed to improve its application and expand the use in NSCLC.

## Introduction

Lung cancer is the second-most diagnosed cancer worldwide and the leading cause of cancer-related mortality. Non-small cell lung cancer (NSCLC) is the most frequent subtype, accounting for 85% of cases. Despite advances in early diagnosis and local and systemic treatments, overall survival is still dismal, with 5-year survival rates of about 10–30% for metastatic patients [1, 2].

Immune checkpoint inhibitors (ICIs) represent a major treatment advance for non-oncogene-addicted NSCLCs, which represent the majority of diagnosed cases. However, only 15 to 30% of people will derive long-term benefit from these treatments given alone or in combination with chemotherapies or other ICIs [3–6]. In fact, 80% of people will develop a primary or secondary resistance to immunotherapy, attributable to several factors, including the insufficient presence of antitumor T cells (“cold tumors”), disruption of antigen presentation machinery, emergence of new inhibitory checkpoints, and impaired formation of memory T cells [7, 8]. Therefore, new therapeutic options are urgently needed.

**Fig. 1:**
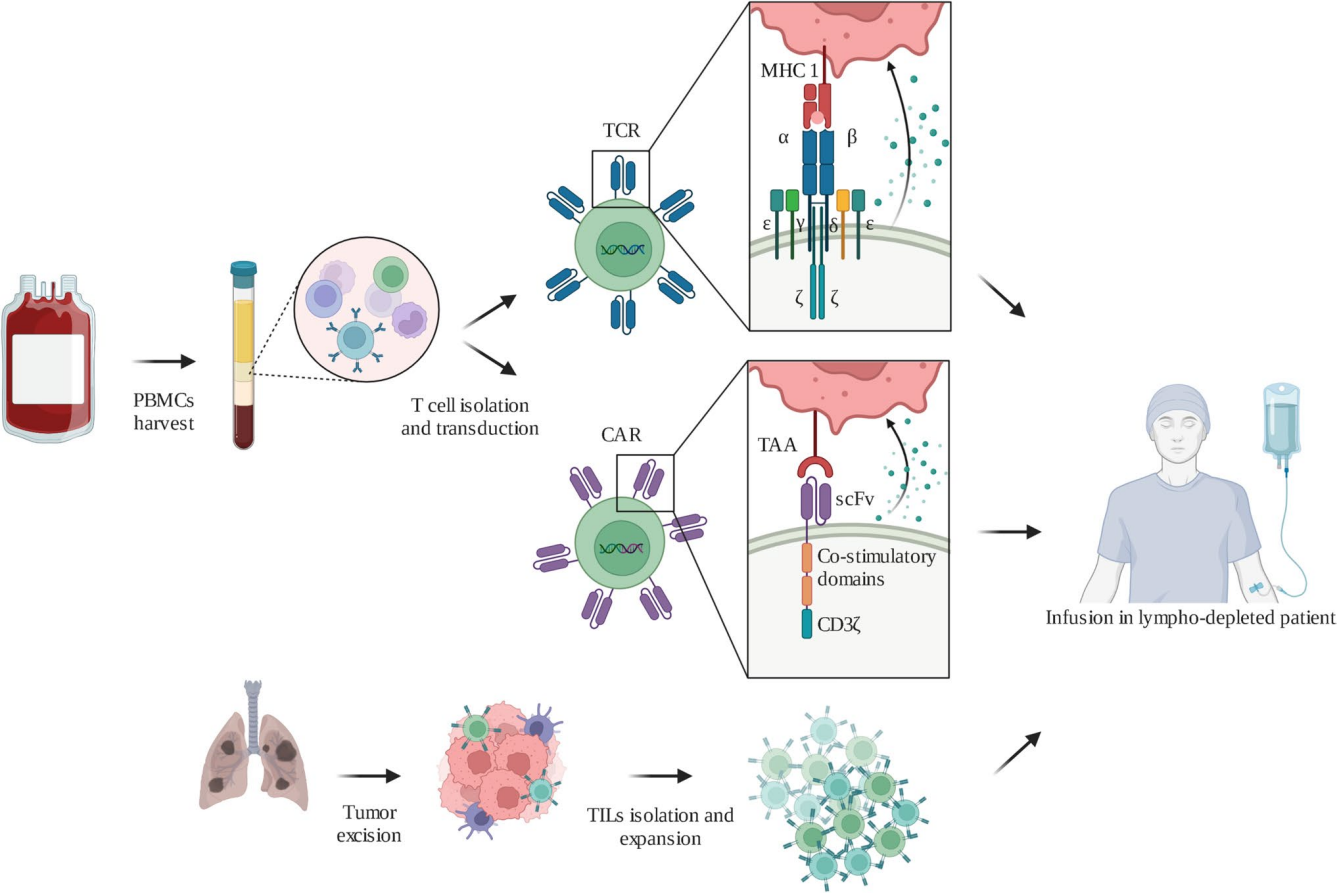
Adoptive cell therapies manufacturing process. For engineered ACT, PBMCs are obtained from autologous peripheral blood. CARs or TCRs are added through transduction. In the case of TILs, lymphocytes are obtained from tumor samples. T cells are expanded in vitro before being infused intravenously into the patients. Figure created with BioRender.com

Cellular therapies, also known as adoptive cell therapies (ACT), are personalized immunotherapies that involve the generation of artificial tumor-reactive T cells, such as engineered T cells expressing transgenic T cell receptors (TCR) or chimeric antigen receptors (CAR), or the infusion of ex vivo expanded endogenous T cells such as tumor-infiltrating lymphocytes (TILs) (Fig. 1) [9]. ACTs have become promising strategies for cancer treatment, mainly in hematologic malignancies, although their application in the management of metastatic NSCLC (mNSCLC) is still under investigation.

In this review, we will provide an overview of different ACT modalities and their evolving roles in mNSCLC. We will also highlight challenges pertaining to the use of ACT and strategies to address these obstacles.

## Engineered ACT

Genetically engineered T cell therapies involve the isolation of autologous T lymphocytes from patients’ peripheral blood, via leukapheresis. Such processes avoid the need for surgery for T cells collection and overcome the limitation of harvesting intratumoral TILs, making it applicable to “cold” tumors.

Antigen-specific TCR or CAR are added to T cells principally through retro- or lentiviral transduction which is highly efficient but personalized and costly, limiting its accessibility. Furthermore, it is impossible to control the site of integration of the CAR or TCR-encoding nucleic acid, increasing the risk of insertional mutagenesis; furthermore, viral vectors could cause infection. More recently, new non-viral techniques such as transposome-based transfection (i.e., CRISPR-Cas9, Sleeping Beauty, and piggyBac) or electroporation have been introduced. Their increasing efficiency, low cost, and possibility of controlling the site of integration make the delivery of genetic information more stable, effective, and sustainable.

Engineered T cells are then expanded in vitro and reinfused into the patient, following a lymphodepleting chemotherapy. Interleukin 2 (IL-2) can be used to increase in vivo expansion of cells [10].

A major challenge for engineered ACT is the selection of a suitable antigen: the ideal target antigen should be broadly expressed on tumor cells, have no or low expression in healthy tissues, and be sufficiently immunogenic to trigger T cell responses.

There are three main classes of tumor antigens: tumor-associated antigens (TAAs), cancer-germline antigens (CGAs), and tumor-specific antigens (TSAs). TAAs include overexpressed antigens (e.g., Wilms’ tumor antigen 1 (WT1)) and cancer differentiation antigens (e.g., melanoma-associated antigen recognized by T cells (MART-1) and mesothelin). As they are expressed not only by tumor cells but also in some normal cells, targeting them can lead to an increased risk of toxicity in healthy tissues. CGAs are aberrantly expressed in cancer cells, while their expression in normal tissue is restricted to germline cells (e.g., New York esophageal squamous cell carcinoma-1 (NY-ESO-1) and melanoma-associated antigen A (MAGE-A), thereby representing promising targets for ACT. Finally, TSAs, which include viral antigens such as those related to oncogenic HPV and EBV viruses and neoantigens, are encoded in cancer cells but are absent from the genome of healthy cells.

Neoantigens stem from nonsynonymous mutations that accumulate in cancer tissue during carcinogenesis and can be detected by T cells. Despite the fact that complex, individualized methods are required for the identification and selection of reactive cells, immunological targeting of neoantigens represents a safe and promising strategy for treating cancer patients, thanks to its specificity for neoplastic cells.

Driver mutations are particularly interesting as they may be expressed homogeneously by cancer cells and shared among patients within particular disease subtypes. However, most oncogene-addicted NSCLC is characterized by low tumor mutation burden (TMB) and neoantigen expression, as well as several genetic, epigenetic, and immunometabolic features that are responsible for immune exclusion and low responsiveness to conventional immunotherapy. Though still in the early stages of development, ACT may have a highly relevant role in this context.

## TCR-T Cells

Conventional T cells recognize enzymatically cleaved peptides that are presented at the cell surface by MHC molecules (pMHC) through their TCR, a heterodimer comprised of an α and a β chain. Recognition of a specific pMHC by the TCRα/β heterodimer leads to the phosphorylation of immunoreceptor tyrosine-based activation motifs in intracellular regions of the CD3 complex subunits and to the formation of a functional receptor, which initiate T cell activation, proliferation, and effector functions, such as cytokine secretion and cytolysis, through secretion of granzyme and perforine.

For their use in adoptive therapies, TCR-T cells are edited by transducing a specific TCR gene sequence that recognizes an intracellular tumor antigen. TCR optimization can be performed to prevent TCRα/β chains mispairing and increase TCR expression and stability [11, 12].

Intracellular tumor antigens, such as proteins, represent about 85% of cancer-associated antigens, and although TCR-T cells could potentially target any of them, the number of safe and effective identified targets is still limited, and most of the T cells are engineered to recognize only one antigen, limiting their application in solid tumors where heterogeneity is a major challenge.

To date, most clinical trials of TCR-T therapy in mNSCLC are targeting CGAs (Table 1), showing good tolerability but modest efficacy, with only few trials reporting results. In one phase 1 trial with a NY-ESO-1 TCR-T cell, one out of 4 patients with mNSCLC reported a partial response (PR), one a stable disease (SD), and none had severe toxicity [9]. A trial testing MAGE-A10-specific TCR-T cells demonstrated an acceptable safety profile in the absence of off-target toxicities: 28% of patients reported cytokine-release syndrome (CRS) of any grade, with one case of grade 4, reversible with appropriate treatment; 2 patients reported grade 4 pancytopenia, probably related to a higher lymphodepleting regimen. Out of the 11 patients, one had a PR and four reported SD [23•].

**Table 1:** Selected clinical trials using TCR ACT therapy in NSCLC

Target and its overexpression prevalence in NSCLC	NCT#	Phase	Population	Other information	Sponsor
KK-LC-1 30–40% [13, 14]	NCT05483491	I	Solid tumors, including NSCLC, KK-LC-1^+^ (≥ 25% by IHC), HLA-A*01:01	Autologous T cells from peripheral blood followed by high-dose IL-2	Christian Hinrichs
	NCT03778814	I	Solid tumors, including NSCLC, HLA-A11, and available autologous transduced T cells with ≥ 20% expression of targeted TCR sequences determined by flow cytometry and killing of tumor cells ≥ 20% in cytotoxicity assay	Autologous T cells from peripheral blood or tumor given by intravenous, intraarterial, or intratumoral reinfusion	Second Affiliated Hospital of Guangzhou Medical University
MAGE A4/8 30–40% [15]	NCT03247309	I	Solid tumors, including NSCLC, MAGE A4/8^+^, and HLA matched (not stated)	Autologous T cells from peripheral blood	Immatics US, Inc.
	NCT03139370	I	Solid tumors, including NSCLC, MAGE-A3 and/or MAGE-A6^+^, (HLA)-DPB1*04:01	Autologous T cells from peripheral blood	Kite, A Gilead Company
MAGE A10 5–25% AC 34–50% SCC [15–17]	NCT02592577	I	Advanced NSCLC, MAGE-A10^+^, HLA-A*02:01, and/or HLA-A*02:06	Autologous specific peptide enhanced affinity receptor (SPEAR) T cells	Adaptimmune
Neoantigens	NCT04102436	II	Solid tumors, including NSCLC	Autologous T Cells from peripheral blood engineered using the Sleeping Beauty Transposon/Transposase System to express TCR reactive against mutated neoantigens with or without high- or low-dose IL-2	National Cancer Institute (NCI)
	NCT03412877	II	Solid tumors, including NSCLC	Autologous T Cells from peripheral blood genetically engineered to express TCR against neoantigens, with or without Pembrolizumab	National Cancer Institute (NCI)
Selected antigens (KRAS G12D 19% [18], KRAS G12V 15% [18], EGFR E746–A750del 10–15% AC [19], TP53 R175H–Y220C 20–40% [20])	NCT05194735	I/II	Solid tumors, including NSCLC, harboring a tumor mutation and HLA typing combination according to Alaunos’ library	Autologous T cells engineered using the Sleeping Beauty transposon/transposase system to express TCR reactive against neoantigens, followed by IL-2	Alaunos Therapeutics
NY-ESO-1 2–20% [15, 21, 22])	NCT05296564	I/II	Solid tumors, including NSCLC, NY-ESO-1^+^ (by IHC), and HLA-A*02	Autologous T cells from peripheral blood	Hadassah Medical Organization
	NCT02457650	I	Solid tumors, including NSCLC, NY-ESO-1^+^ (by IHC), and HLA-A*02	Autologous T cells from peripheral blood followed by low-dose IL-2	Shenzhen Second People’s Hospital
	NCT03240861	I	Solid tumors, including NSCLC, NY-ESO-1^+^ (by IHC), and HLA-A*0201	Genetically engineered PBMC and PBSC expressing NY-ESO-1 TCR, followed by IL-2	Jonsson Comprehensive Cancer Center

*TCR* T cell receptor, *ACT* adoptive cell therapy, *NSCLC* non-small cell lung carcinoma, *KK-LC-1* Kita-kyushu lung cancer antigen 1, *IHC* immunohistochemistry, *HLA* human leucocyte antigen, *IL* interleukin, *MAGE* melanoma antigen gene, *AC* adenocarcinoma, *SCC* squamous cell carcinoma, *EGFR* epithelial growth factor receptor, *NY-ESO-1* New York esophageal squamous cell carcinoma-1, *PBMC* peripheral blood mononuclear cell, *PBSC* peripheral blood stem cell

Personalized and bioinformatics-driven approaches are being investigated in this field. IMA101 is a personalized multi-target ACT approach in which autologous blood-derived T cell products are redirected against multiple novel defined peptide-HLA cancer targets identified by an outsourced platform from a pool of predefined targets. It is currently being tested in relapsed or refractory solid tumors among patients whose tumors express at least one of the most frequent CGAs (MAGEA1, MAGEA4, MAGEA8, NY-ESO-1, etc.).

Attempts to broaden the TCR strategy may largely depend on the identification and targeting of neoantigens. The next step for these platforms is the identification of high-affinity neoantigens and TCR engineering toward several of these. Clinical trials are currently ongoing to determine the feasibility, safety, and efficacy of these personalized ACTs (Table 1).

It is important to highlight that engineered TCRs are HLA restricted; hence, they require HLA matching to be effective. Many of the products under investigation today are restricted to HLA-A*0201, which is under-represented in African and Asian populations despite being present in up to half of Caucasians [24].

## CAR-T Cells

CAR-T cells have gained lot of attention due to their outstanding results in hematological malignancies. CARs are fully artificial receptors designed to recognize specific antigens without HLA presentation, thus targeting only membrane-bound antigens. Their structure contains an extracellular domain responsible for the antigen recognition, a transmembrane domain, and the intracellular signaling domain. The extracellular domain is composed of a single-chain variable fragment (scFv), which is the major element of the antigen-binding domain, linked to the transmembrane domain by a hinge region. The length of the hinge region can be adjusted to optimize the distance between CAR-T cells and targeted tumor cells for CAR signal transduction. The intracellular domain can contain several functional units, with the core component being a CD3ζ chain, which is responsible for T cell activation.

**Fig. 2:**
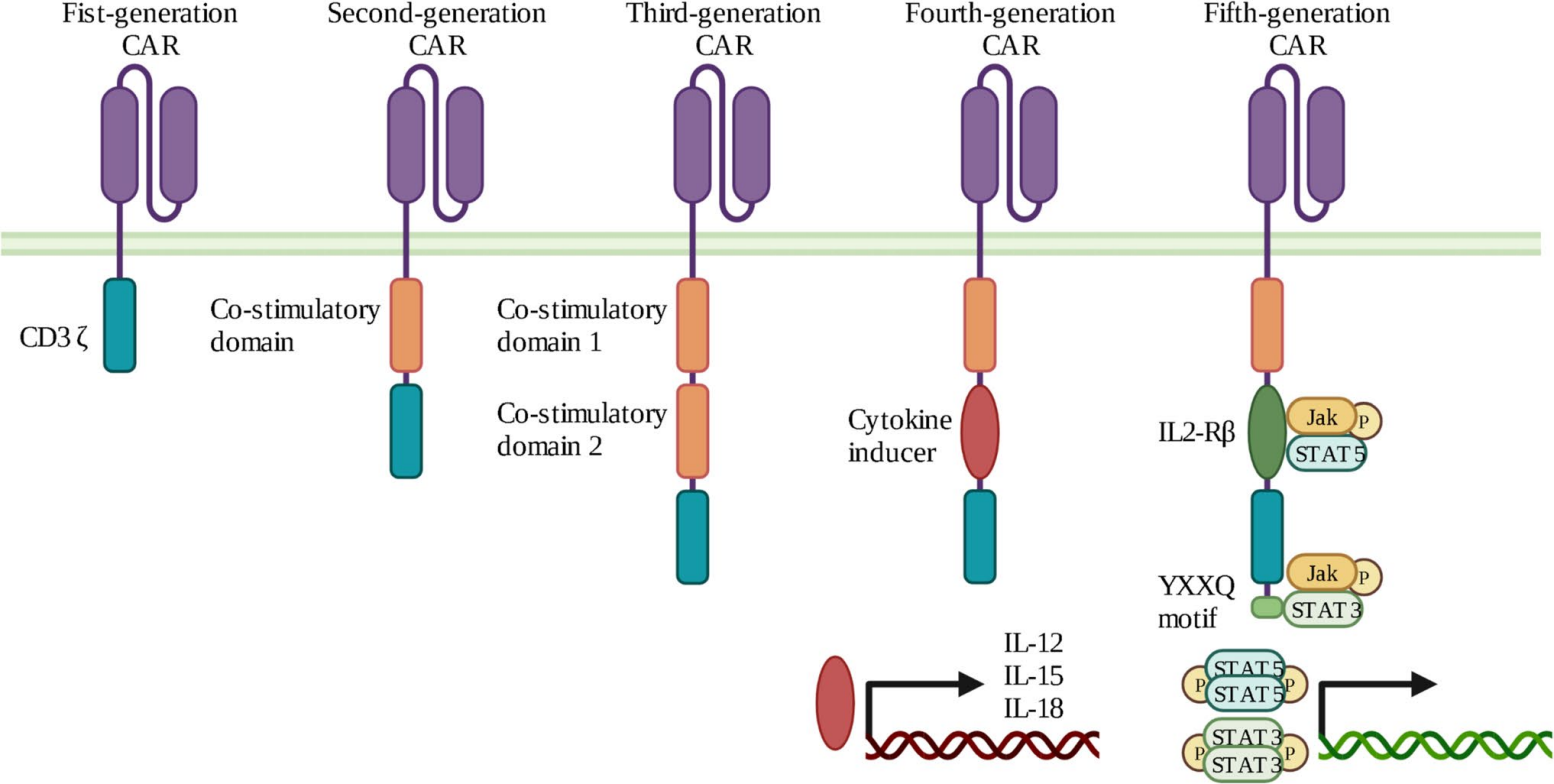
Structure of different CAR-T generations. Compared to the first generation, that contained only one intracellular component, CD3ζ, the second- and third-generation CARs include one or two co-stimulatory domains, respectively. The fourth generation of CARs is based on second generation with the addition of an inducibly expressed chemokine. The fifth generation is characterized by the incorporation of a truncated cytoplasmic domain of IL-2Rβ for STAT5 recruitment and a STAT3-binding YXXQ motif allowing activation of the JAK/STAT signaling after antigen engagement

Different generations of CARs have been developed, differing by the structure of their intracellular domain (Fig. 2). First-generation CARs only had a CD3ζ binding site, leading to insufficient activation signal. Second- and third-generation CARs were generated by adding one or two co-stimulatory domains, respectively, to increase proliferation and cytotoxicity, such as 4-1BB (also known as CD137) and CD28. Interestingly, 4-1BB and CD28 are not equivalent. It has been suggested that 4-1BB favors T cell memory-associated genes, while CD28 leads to an exhausted phenotype more quickly [25]. Fourth-generation CARs are also called “T cells redirected for antigen-unrestricted cytokine-initiated killing” (TRUCKs). They are additionally engineered to secrete a transgenic cytokine upon CAR signaling in the targeted tumor tissue, such as IL-12 or IL-15, to improve persistence and cytotoxicity [26].

Fifth-generation CARs are currently under development and differ from previous versions for the integration of an additional membrane receptor. In fact, they contain a truncated cytoplasmic IL-2 receptor β-chain domain with a binding site for the transcription factor STAT3. Antigen binding to this receptor leads to simultaneous activation of triple signaling by CD3ζ, costimulatory molecules, and the JAK–STAT3/5 pathway, improving T cell activation, proliferation, and persistence. The fifth-generation CARs are also manufactured to have a better safety profile and a wider therapeutic window but are still limited by issues of tumor trafficking and toxicities.

Several surface antigens in NSCLC such as epidermal growth factor receptor (EGFR), carcinoembryonic antigen (CEA), human epidermal growth factor receptor 2 (HER2), mesothelin (MSLN), disialoganglioside (GD2), receptor tyrosine kinase-like orphan receptor 1 (ROR1), mucin 1 (MUC1), glypican-3 (GPC3), delta-like ligand 3 (DLL3), and PD-L1 are currently under investigation for CAR-T therapeutics (Table 2). Most of the trials are histology-agnostic phase I trials and include heterogeneous cohorts of patients diagnosed with different tumor types that share the expression of a common surface protein. Only a minority of CAR-T clinical trials in mNSCLC patients have reported preliminary results, describing mostly modest clinical activity (Table 2).

**Table 2:** Selected clinical trials using CAR-T ACT therapy in NSCLC

Target and its overexpression prevalence in NSCLC	NCT#	Phase	Population	Other information	Sponsor
CLDN6 40% [27]	NCT04503278	I/II	Solid tumors, including NSCLC, CLDN6^+^ (≥ 50% of tumor cells expressing ≥ 2^+^ CLDN6 protein by IHC)	CLDN6 CAR-T with or without CLDN6 RNA-LPX cancer vaccine	BioNTech Cell & Gene Therapies GmbH
CEA 50% [28]	NCT04348643	I/II	Solid tumors, including NSCLC, CEA^+^ (by IHC) or serum CEA level >50 ng		Chongqing Precision Biotech Co., Ltd
	NCT05736731	I	Solid tumors, including NSCLC, CEA^+^ (by IHC), and HLA-A*02 loss	Autologous logic-gated Tmod™ CAR-T cell product	A2 Biotherapeutics Inc
EGFR 40–90% [29]	NCT01869166	I	NSCLC, EGFR^+^ (>50% IHC expression)	Autologous lentivirus transduced CAR-T cells	Chinese PLA General Hospital
	NCT03182816	I	NSCLC, EGFR^+^ (>50% IHC expression)	Autologous piggyBac transposon system generated CAR-T cells; second part with anti-PDL1 and anti-CTLA4 secreting EGFR CAR-T cells	NCT03182816
	NCT05060796	I	NSCLC, EGFR^+^ (≥2+ by IHC); CXCL13 factor positive rate ≥ 10%	CXCR5 modified EGFR CAR-T cells	Second Affiliated Hospital of Guangzhou Medical University
GPC3 55% SCC [30]	NCT05120271	I/II	SCC-NSCLC with GPC3 overexpression (by IHC)		SOTIO, LLC
ROR1 60% [31]	NCT02706392		NSCLC, ROR1^+^ (>20% by IHC)	Autologous 41BB CAR-T	Fred Hutchinson Cancer Center
	NCT05274451	I	NSCLC, ROR1^+^ (by IHC)		Lyell Immunopharma, Inc.
MSLN 30–70% AC [32]	NCT02414269	I/II	NSCLC metastatic to the pleura, MSLN^+^ (>10% by IHC), or elevated serum SMRP levels (>1.0 nM/L)	Intrapleural injection of iCasp9M28z CAR-T cells	Memorial Sloan Kettering Cancer Center
B7H3 35% [33]	NCT05190185	I	NSCLC, B7H3^+^ (≥1% by IHC)		PersonGen BioTherapeutics (Suzhou) Co., Ltd.
Several (HER2 13–20% [34], MSLN, MUC1, GPC3, EGFR, B7-H3)	NCT03198052	I	Solid tumors, including NSCLC, target-positive (by IHC)	Autologous transduced T cells with > 20% expression of target-antigen CAR determined by flow cytometry and killing of target-positive cells > 20% in cytotoxicity assay	Second Affiliated Hospital of Guangzhou Medical University

*CAR* chimeric antigen receptors, *ACT* adoptive cell therapy, *NSCLC* non-small cell lung carcinoma, *CLDN6* Claudin-6, *IHC* immunohistochemistry, *CEA* carcinoembryonic antigen, *HLA* human leucocyte antigen, *EGFR* epithelial growth factor receptor, *PDL1* programmed cell death lidang-1, *CTLA4* cytotoxic T-lymphocyte-associated protein 4, *CXCR* C-X-C motif chemokine receptors, *GPC3* Glypican 3, *ROR1* retinoic acid-related orphan receptor-1, *SCC* squamous cell carcinoma, *MSLN* mesothelin, *AC* adenocarcinoma, *SMRP* soluble mesothelin-related peptides, *HER2* human epidermal growth factor receptor-2, *MUC1* Mucin-1

Two phase I clinical trials demonstrated the safety of EGFR-targeting CAR-T cell in EGFR-overexpressing relapsed/refractory mNSCLC. In the first trial, two out of 11 patients achieved a partial response (PR) [35•]. In 4 patients who underwent biopsies after CAR-T cell treatment, there was a pathological eradication of EGFR-positive tumor cells, and CAR-EGFR genes were detected in TILs. In the second trial, among nine patients treated with a non-viral transposon-based gene transfer system of EGFR CAR, one patient achieved durable PR that persisted for more than 1 year [36•]. Both trials showed good CAR-T expansion in most of the patients.

EGFR is an antigen expressed in both epithelial cancers and healthy epithelial cells; thus, its targeting has raised many concerns as regards on-target off-tumor toxicities. However, both trials reported mainly grade 1–2 toxicities (mostly skin rash and dyspnea), and only 2 grade 3 toxicities were reported (increase in pancreatic enzymes and fever), both manageable and reversible. These experiences show that fine tuning of epitope affinity of CAR may overcome this issue, allowing the preferential recognition of target in higher-level expressing tumoral cells, although more studies are needed to confirm this hypothesis. A phase I trial of MUC1-targeted CAR-T cells with PD-1 knockout through CRISPR-Cas9 for the treatment of mNSCLC patients had manageable toxicity with no grade ≥ 3 adverse events. However, no signs of activity were detected, with ORR of 0% and 55% (11/20) of the patients experiencing SD as the best response [37]. Early results of a phase I clinical study demonstrated the safety of anti-ROR1 CAR-T cells in mNSCLC patients. Yet, only a mixed response was observed in the two mNSCLC patients included [38].

HLA class I alterations are the cause of about 30% of cases of resistance to ICIs [8••], making CAR-T independency from HLA presentation an attractive prospect for NSCLC. The lack of an HLA subtype constraint would also expand the target population; however, due to disease heterogeneity and the abundance of intracellular antigens, restricting CAR recognition to a single and superficially expressed antigen is not ideal in advanced NSCLC.

## Endogenous ACT

### Tumor-Infiltrating Lymphocytes (TILs)

TIL-based ACT involves the infusion of a large number of cultured cells derived from patient’s TILs, necessitating surgery to gather enough material. Cells are cultured in the presence of IL-2 and feeder cells and reinfused to the patient after a preparatory course of high-dose non-myeloablating chemotherapy, typically consisting of cyclophosphamide and fludarabine. Subsequently, medium- to high-dose IL-2 is administered to the patient in order to increase the T cells’ in vivo expansion and efficacy.

TILs’ products are highly polyclonal, resulting in a multi-target T cell attack directed against different and largely unknown antigens. In addition, in vivo expansion of TILs can release cells from an immunosuppressive microenvironment and reactivate them to target tumors.

Most trials were conducted with “bulk” unselected TILs, but significant efforts are being made to improve tumor selectivity. Bulk TILs have shown durable responses in subsets of metastatic melanoma (MM) patients pretreated with approved ICIs. In a recent randomized phase II study, TILs were compared to ipilimumab in patients with diseases refractory to anti-PD1 treatment, showing significantly higher progression-free survival and overall survival and 30% of complete response (CR), which normally correlate with long-term responses to treatment [39••]. Most epithelial tumors have lower T cell infiltration than MM, and their T cells are more challenging to expand in vivo. Additionally, the high tumor mutational burden (TMB) and emergence of neoantigens, as well as a far less immunosuppressive microenvironment in MM than NSCLC, are all related to the success of TIL-based ACT in MM. Nonetheless, TIL-based ACT is supported by a growing body of evidence in NSCLC. The presence of neoantigen-responsive endogenous CD4 and CD8 T cells in wild-type disease and recurrent oncogenic mutations offers hope [40, 41].

Bulk TILs have been investigated in a phase Ib study in patients affected by mNSCLC [42••]. Twenty patients were enrolled regardless of PDL1 expression, TMB, smoking status, and the presence of actionable mutations; 4 patients harbored an EGFR mutation (2 of which were activating, and 2 an ALK translocation). Half of the patients had not received any systemic treatment; 20% were chemotherapy naïve; and all were immunotherapy naive. Patients were treated within the trial with nivolumab in monotherapy for at least 4 cycles. Sixteen out of 20 patients progressed on anti-PD1 monotherapy and proceeded to receive TIL treatment. Most of the patients (11/16) experienced tumor regression at 1 month but then progressed, mainly with the appearance of new lesions, indicating a possible resistance due to subclonal selection and antigen escape. Two patients reported CR and were still in response at the time of the publication. Interestingly, CR was observed in a patient whose tumor harbored an EGFR-activating mutation. The analysis of the product she received showed that her TIL clonotypes recognized a private neoantigen and several MAGE-associated TAAs. The same group is conducting a phase I trial in immune-naïve oncogene-addicted NSCLC with CD40L-expressing TILs and nivolumab (NCT05681780). Toxicity was consistent with previous results, mainly related to lymphodepleting chemotherapy (hematologic toxicities) and high-dose IL-2 (chills, fever, and capillary leak syndrome). Most of the adverse events were limited to the first 4 weeks after infusion. The authors reported 2 toxic deaths related to high age and comorbidities, underlying the importance of patient selection.

Selection of neoantigen-reactive TILs in solid tumors is being investigated in a number of trials (Table 3). The Chiron trial is a phase I/IIa study aiming to characterize the safety and clinical activity of a personalized clonal neoantigen-reactive T cell (cNeT) product in mNSCLC, progressing after an anti-PD1/PDL1-based treatment. The trial excluded non-smokers and patients with actionable mutations. Clonal neoantigens emerge early in cancer evolution and are likely to be shared by most of cells, limiting the effects of tumor heterogeneity and antigen escape [43••]. Furthermore, manufacturing of cNeT product involves the use of dendritic cells cultured with low doses of IL-2, resulting in greater IL-2 responsiveness in patients, allowing product infusion after lower dose lymphodepletion and IL-2, in order to limit associated toxicities and broaden applicability, even in the outpatient setting. The resulting treatment was well tolerated, with lower chemotherapy and IL-2-associated adverse events. Early proof of concept was demonstrated in mNSCLC with disease control observed at more than 12 weeks in 5 of 7 evaluable patients (71%), including one PR ongoing at 36 weeks after the treatment.

**Table 3:** Selected ongoing clinical trial using TILs ACT in NSCLC

NCT#	Phase	Population	Other information	Sponsor
NCT05681780	I/II	EGFR, ALK, ROS1 or HER2-driven NSCLC, PD1-PDL1 naive	CD40L-augmented autologous TILs, given in combination with IL-2 and nivolumab	H. Lee Moffitt Cancer Center and Research Institute
NCT05676749	I	NSCLC without driver mutations, no previous anti-PD1/PDL1 unless given for locally advanced disease and > 6 months before enrollement	Autologous TILs (C-TIL051) given in combination with IL-2 and pembrolizumab	Cellular Biomedicine Group, Inc.
NCT04614103	I	NSCLC without EGFR, ALK, ROS1 alterations, refractory to stardard platinum based/ICI treatment	Autologous TILs (LN-145) followed by high-dose IL-2	Iovance Biotherapeutics, Inc.
NCT02133196	II	NSCLC (including oncogene-addicted) progressing after first line therapy	Young TILs followed by high-dose IL-2	NCI
NCT05361174	I	NSCLC (including oncogene-addicted), progressing within 12 weeks after PD1 based therapy	Genetically modified autologous TILs (disruption of PDCD1, PD-1 gene) followed by IL-2	Iovance Biotherapeutics, Inc.
NCT03645928	II	NSCLC (including oncogene-addicted) PD1/PL1 naive or pretreated (according to cohort)	Autologous TILs LN-144 (lifileucel)/LN-145 in combination with high-dose IL-2 and checkpoint inhibitors (ipilimumab/nivolumab or pembrolizumab) or autologous TILs LN-144 (lifileucel)/LN-145/LN-145-S1 as a single agent therapy followed by IL-2	Iovance Biotherapeutics, Inc.
NCT04643574	I	NSCLC (including oncogene-addicted) progressing after first line therapy	Autologous TILs enriched for tumor antigen specificity (NeoTIL) in combination with low dose irradiation and high-dose IL-2	Centre Hospitalier Universitaire Vaudois
NCT05141474	I	Solid tumors, including NSCLC, progressing after ICIs	Neoantigen-selected autologous TILs (NEXTGENTIL-ACT) in combination with IL-2	Vall d’Hebron Institute of Oncology
NCT04032847	I	NSCLC, smokers, without actionabla mutations, progressing after ICIs	Autologous clonal neoantigen-reactive T cells (cNeT) followed by low-dose IL-2, in monotherapy or in combination with pembrolizumab	Achilles Therapeutics UK Limited

*TILs* tumor-infiltrating lymphocytes, *ACT* adoptive cell therapy, *NSCLC* non-small cell lung carcinoma, *EGFR* epithelial growth factor receptor, *ALK* anaplastic lymphoma kinase, *HER2* human epidermal growth factor receptor-2, *PD1* programmed cell death-1, *PDL1* programmed cell death ligand-1, *ICI* immune checkpoint inhibitors

#### Challenges and Perspectives

**Fig. 3:**
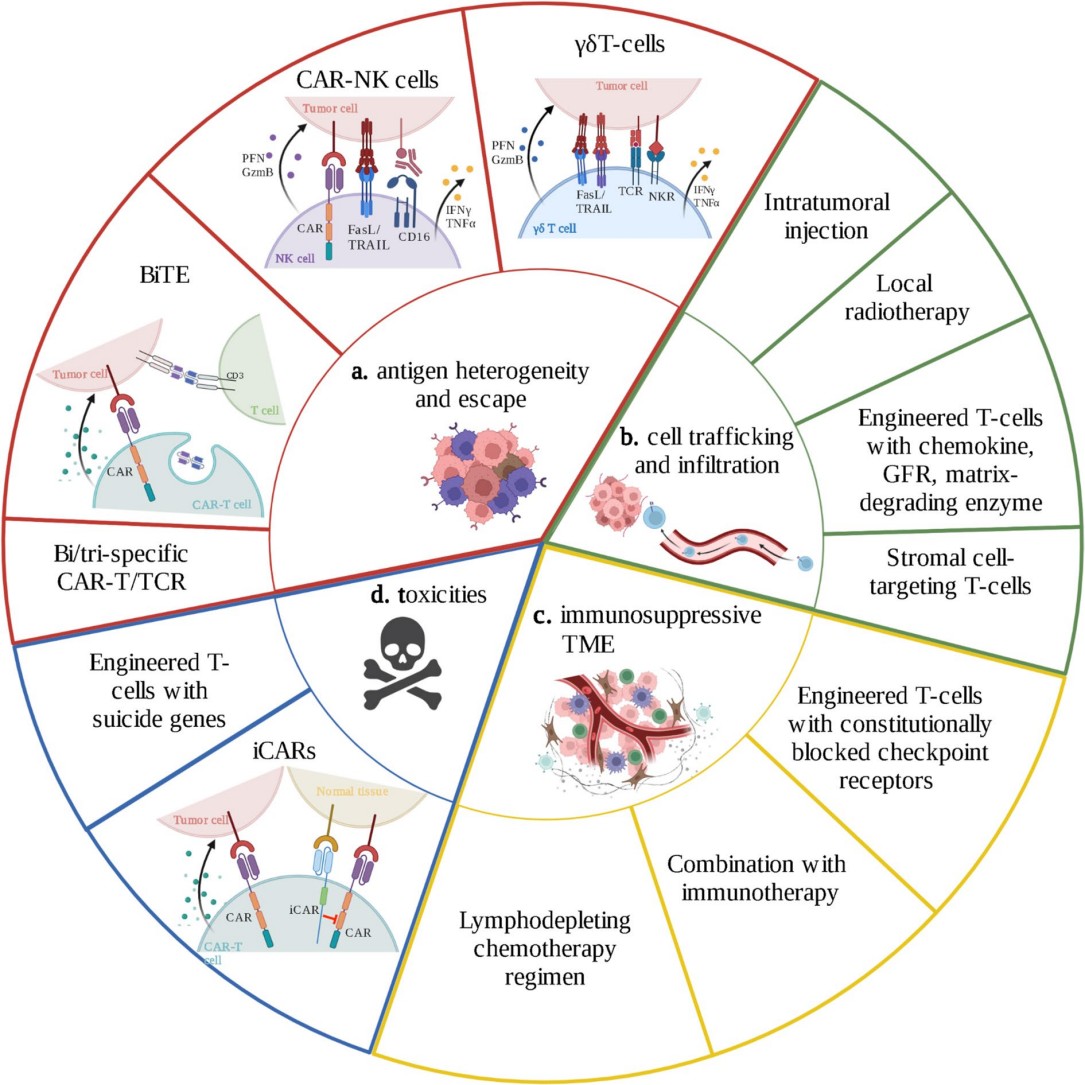
Challenges and perspectives of ACT in NSCLC. **a** Several approaches are being developed to overcome tumor antigen heterogeneity and escape, such as CAR-T cells or TCR endowed with specificity for multiple targets or expressing bispecific T cell engagers. The use of NK cells or γδT cells for ACT represents a promising strategy as these cells are independent of MHC-presentation, depict innate immune activities, and, by secreting IFNγ and TNFα, stimulate bystander T cells. **b** Immune cell trafficking and penetration are limited in solid tumors. Cells can be developed with the ability to respond to tumor-associated chemokines or to target physical barriers present in the tumor microenvironment. Alternatively, immune cells can be directly injected into the site of the tumor. Stroma modulation with local radiotherapy can also improve immune cell infiltration. **c** In order to counteract the immunosuppressive TME, T cells can be engineered with constitutionally blocked checkpoint receptors. Combination with ICI or lymphodepletive chemotherapy is another approach to remodel the TME. **d** To overcome on-target, off-tumor toxicities of CAR-T cells, the tumor specificity can be improved by ensuring dependency of activation on the absence of an antigen selectively expressed on non-tumoral cells. The suicide gene system allows the elimination engineered cells via induction of apoptosis in case of systemic toxicity. CAR, chimeric antigen receptor; TCR, T cell receptor; BiTE, bispecific T cell engager; PFN, perforin; GzmB, granzyme B; FasL, Fas ligand; TRAIL, tumor-necrosis-factor related apoptosis inducing ligand; IFNγ, interferon γ; TNFα, tumor necrosis factor α; GFR, growth factor receptor; TME, tumor microenvironment; iCAR, inhibitory chimeric antigen receptor; ACT, adoptive cell therapy; NSCLC, non-small cell lung carcinoma; ICI, immune checkpoint inhibitors. Figure created with BioRender.com

The extensive clinical adoption of T cell-based ACT in solid tumors is being constrained by considerably more therapeutic hurdles compared to hematological malignancies. Target antigen selection, antigen escape mechanisms, T cell homing and tumor infiltration, the immunosuppressive tumor microenvironment (TME), toxicities, and manufacturing difficulties are some of these challenges (Fig. 3).

### Antigen Heterogeneity and Escape

Solid tumors show significant inter- and intra-patient heterogeneity in antigen expression [44]. Immune escape through antigenic loss is a common problem of T cell therapies and results in tumor recurrence [45, 46].

As discussed, TILs are highly polyclonal by nature and further selection of clonal neoantigens and tumor-reactive clones could improve their efficacy. One solution for engineered ACTs could be to target multiple antigens by using T cell clones with different tumor-specific TCRs or CARs. Bi- or trispecific CAR-T cells are being tested in solid tumors [47–50]. By equipping TCR-T cells and CAR-T cells with bispecific T cell engagers (BiTEs), it is possible to achieve the same effect by activating bystander T cells as well as the endogenous immune system [51, 52].

The further development of ACT or their combination with immunomodulatory drugs, in order to facilitate tumor debulking and release of antigens, followed by the activation of an endogenous response to secondary tumor antigens, is another potential tactic to oppose this tumor escape mechanism. This phenomenon, known as epitope spreading, has been shown to be promoted by CAR-T cells or TILs (NCT05681780) expressing cytokines or CD40L as well as T cells secreting the FLT3 ligand [53–55].

### HLA Loss

Loss of HLA expression or defects in the antigen-processing machinery are a frequent escape mechanisms in NSCLC and are associated with decreased T cell infiltration [8, 46, 56, 57].

In particular, HLA-class I loss of heterozygosity (LOH) is a marker that discriminates cancer from healthy cells and could be exploited for cancer immunotherapy for increasing killing selectivity [58].

Indeed, Tmod^TM^ are logic-gated CAR-T cells engineered to express 2 receptors, one being an activator that recognizes an antigen present on the surface of healthy and tumor cells, and one being a blocker that recognizes a second surface antigen from an allele lost only in tumor cells. This creates a robust and non-signal integrator capable of killing tumor cells while leaving healthy cells intact and thus potentially reducing toxicity [59]. This technology is being tested in CEA-expressing tumors with HLA-A02 LOH (NCT05736731).

One other possible strategy to overcome this barrier is the use of different immune cells that do not require HLA expression to exert their activity, like NK and γδT cells. NK cells are naturally cytotoxic against cancer and virus-infected cells and are not restricted by MHC. Compared to T cells, they demonstrate several advantages: NK cells are less toxic as they do not trigger cytokine-release syndrome, and they retain antitumoral effects through their innate cytotoxic activity in case of tumor escape through antigen loss [60]. Moreover, they offer the possibility of “off-the-shelf” manufacturing as allogeneic NK cells do not cause graft-versus-host disease [61]. CAR-NK and TCR-NK cells are under development with early trials showing promising results [62, 63]. γδT cells represent a small subset of CD8-positive T cell displaying both innate- and adaptive-like properties [64]. γδTcells share many characteristics with their αβT cell counterpart, such as cytotoxic effector functions, but express a distinct TCR composed of a γ and a δ chain that is independent from MHC-I presentation of antigens. Furthermore, these cells can be activated by several innate receptors such as NKG2D, DNAM-1, NKp30, or NKp44 [65]. They can be modified using engineering techniques [66], but similarly to NK cells, allogenic products have a very low risk of GVHD, allowing off-the-shelf formulations. In a recent trial, authors demonstrated safety and preliminary clinical benefit of allogenic γδT cells in mNSCLC [67].

### T Cell Trafficking/Infiltration

T cell migration into tumors is dictated not only by chemokines and adhesion molecules, but also by the immunomodulating tumor stroma that is characterized by a highly dense extracellular matrix and abnormal vasculature.

All strategies intended to modulate tumor stroma, such as low-dose or high-dose radiotherapy or antiangiogenic drugs, could be potentially combined with ACT (NEOTIL), although specific T cell engineering with chemokines, growth factor receptors, and matrix degrading enzymes could obtain the same results in a more tumor-specific fashion, thereby also limiting adverse events. Redirecting of T cells against stromal cell-associated antigens in addition to classic tumor targeting could also improve local delivery. Such strategies are being investigated in solid tumors in the context of TIL-based ACT and CAR-T [68, 69].

One solution may be an intratumoral injection of ACT or part of T cell products. This has been effective in brain tumors and mesothelioma [70–72], but could be more challenging in intraparenchymal lung lesions and in cases of high tumor burden.

### Immunosuppressive TME and T Cell Exhaustion

Solid tumor cells are intermixed with suppressive cell populations such as tumor-associated macrophages (TAMs), myeloid-derived suppressor cells (MDSCs), Tregs, and cancer-associated fibroblasts (CAFs). Tumor and TME cells express a broad range of immune checkpoints, including PD-L1 and ligands for LAG-3, TIM-3, and TIGIT, and the emergence of new immune checkpoints and late exhaustion are common resistance mechanisms to immunotherapies.

Combination with ICIs is one of the most studied strategies, with most of new trials with TILs, CAR-T, and TCR studying the association of ACT with PD1/PDL1 blockade (NCT04032847, NCT03645928). Such an association has a strong scientific rationale since the efficacy of ACT might be compromised by the expression of suppressive immune checkpoints, and conversely, immune checkpoint blockers alone might not have an effect in the absence of sufficient immune effector cells. T cells can also be engineered to have constitutionally blocked checkpoint receptors or allow intratumoral secretion of specific molecules to obtain the same results (NCT05361174).

Lymphodepletive chemotherapy is used because it alters and polarizes the TME, has cytotoxic effects on the host lymphoid population, induces the cytokine sink necessary for adoptive cell proliferation, and depletes some populations of immunosuppressive cells. Further research should be done on the use of other chemotherapies like gemcitabine, taxanes, and some platinum compounds with ACTs because they are linked to immunostimulatory changes in the TME [73].

### Manufacturing Issues

Manufacturing autologous ACT can be challenging due to invasive procedures, and in particular, the need for surgical procedures for TILs-based ACT is cause of significant patient selection and risk for significant morbidity.

Moreover, prolonged production times prevent some patients from receiving the product due to disease progression; this is even more true in the context of personalized approaches that necessitate the processing and analysis of tumors with complex platforms.

In this context, “off-the-shelf” approaches are appealing and include the use of allogeneic cell-based products. While T cell-based approaches can be limited by significant risks of GVHD, NK, or γδ-based products harbor a significantly lower risk, as already discussed.

In this context, a very attractive alternative to cellular therapies is the redirection of T cells through the use of bispecific compounds. Bispecific antibodies can target surface-expressed tumor antigens and one effector cell antigen (such as CD3 for lymphocytes or CD56 for NK cells) and exploit bystander immune cells in order to target tumor cells in a MHC-independent fashion, similarly to CAR-engineered cells. To target various antigens, the number of antigen-recognition domains can be increased while modifying affinities and efficacy. Similarly, it is possible to create and employ TCR-based bispecifics to target intracellular antigens shown by the MHC complex. Tebentafusp is approved by the FDA for the treatment of metastatic uveal melanoma and proves that this approach can be effective in the context of solid tumors.

These methods, however, rely on the presence of endogenous immune cell infiltration, making them potentially ineffective in the event of cold tumors.

### Toxicities

Engineered ACT can elicit potent immune responses but are at risk of inducing “off-target” and “on-target, off-tumor toxicities”, respectively, linked to cross-reactivity or expression of tumor-associated antigens in normal tissues. This makes antigen selection key in the development of such products.

Using autologous rather than engineered TILs reduces the risk of these side effects but requires stringent patient selection, as patients must be fit enough to receive high-dose chemotherapy and IL-2.

There are many ways to decrease adverse effects, such as limiting CAR activity to tumors and incorporating inhibitory CARs (iCAR) into T cells to reduce toxicity to healthy tissue. iCAR consists of an scFv specific to antigens expressed only in normal cells, with potent acute inhibitory signaling to restrict T cell activation despite concurrent engagement of the activating receptor. Safety genes can also be added into the construct. They are also called suicide genes and code for molecules expressed on CAR-T cells (or less frequently on TILs or TCR-engineered T cells) that lead to their death upon administration of a specific drug. Administration of a synthetic molecule induces the dimerization of the chimeric protein coded by the safety gene, which induces apoptosis.

## Conclusion

In this review, we highlight the most recent developments in ACT for the treatment of mNSCLC, examining the benefits, present challenges, and innovative approaches the near future may hold.

There has been significant progress, thanks to advancements in the underlying science and production techniques, and numerous strategies that take advantage of various immune cell types are currently being studied. Promising findings from early-phase studies offer a theoretical basis for their application in mNSCLCs resistant to conventional therapies. However, as resistance mechanisms vastly differ among patients, personalized strategies should be used to tailor the best ACT strategy for the right patient and ensure that it can be used in routine practice.

Significant translational and early- and late-phase clinical research are required before these treatments can be made available to patients with mNSCLC. Currently, many hurdles still exist, including biological or fitness restrictions, optimizing therapeutic efficacy, further understanding the implications of combination treatments, and reducing costs and toxicities.
